# Genome-Wide Identification of Myxobacterial Predation Genes and Demonstration of Formaldehyde Secretion as a Potentially Predation-Resistant Trait of *Pseudomonas aeruginosa*

**DOI:** 10.3389/fmicb.2019.02650

**Published:** 2019-11-13

**Authors:** Daryn Sutton, Paul G. Livingstone, Eleanor Furness, Martin T. Swain, David E. Whitworth

**Affiliations:** ^1^Institute of Biological, Environmental and Rural Sciences, Aberystwyth University, Aberystwyth, United Kingdom; ^2^Department of Biomedical Sciences, Cardiff Metropolitan University, Cardiff, United Kingdom

**Keywords:** public-goods predation, comparative genomics, prey resistance, formaldehyde dismutase, soil ecology

## Abstract

Despite widespread use in human biology, genome-wide association studies (GWAS) of bacteria are few and have, to date, focused primarily on pathogens. Myxobacteria are predatory microbes with large patchwork genomes, with individual strains secreting unique cocktails of predatory proteins and metabolites. We investigated whether a GWAS strategy could be applied to myxobacteria to identify genes associated with predation. Deduced proteomes from 29 myxobacterial genomes (including eight *Myxococcus* genomes sequenced for this study), were clustered into orthologous groups, and the presence/absence of orthologues assessed in superior and inferior predators of ten prey organisms. 139 ‘predation genes’ were identified as being associated significantly with predation, including some whose annotation suggested a testable predatory mechanism. Formaldehyde dismutase (*fdm*) was associated with superior predation of *Pseudomonas aeruginosa*, and predatory activity of a strain lacking *fdm* could be increased by the exogenous addition of a formaldehyde detoxifying enzyme, suggesting that production of formaldehyde by *P. aeruginosa* acts as an anti-predation behaviour. This study establishes the utility of bacterial GWAS to investigate microbial processes beyond pathogenesis, giving plausible and verifiable associations between gene presence/absence and predatory phenotype. We propose that the slow growth rate of myxobacteria, coupled with their predatory mechanism of constitutive secretion, has rendered them relatively resistant to genome streamlining. The resultant genome expansion made possible their observed accumulation of prey-specific predatory genes, without requiring them to be selected for by frequent or recent predation on diverse prey, potentially explaining both the large pan-genome and broad prey range of myxobacteria.

## Introduction

Predatory microbes are increasingly being recognised as apex predators, shaping the population structure of microbial ecosystems (Zhang and Lueders, 2017). They are also prospected and exploited to provide novel therapies for fighting microbial infections (Weissman and Müller, 2010; Findlay, 2016). Myxobacteria are ‘public-goods’ predators, secreting antimicrobial substances into the environment, and leading to the dubious description of their predation as cooperative (Marshall and Whitworth, 2019). Nevertheless, myxobacteria exhibit broad prey ranges – being active against Gram-negative bacteria, Gram-positive bacteria and fungi (Morgan et al., 2010; Livingstone et al., 2017, 2018b). Predation involves the secretion of both secondary metabolites and proteins, which can be packaged into outer membrane vesicles (OMVs) for delivery to/into prey cells (Evans et al., 2012; Berleman et al., 2014; Whitworth et al., 2015). Despite being able to sense chemicals secreted by prey (Lloyd and Whitworth, 2017), predatory cells seem to constitutively secrete cocktails of toxins, independently of whether/which prey is present (Evans et al., 2012; Livingstone et al., 2018a), and this presumably explains their broad prey range.

However, the myxobacterial pan-genome is extremely large, with substantial differences in gene sets between strains of the same genus. A recent study found that 5,600 of the 8,000 genes in a typical *Corallococcus* sp. strain belong to the accessory rather than the core pan-genome, and 75% of genes of the accessory pan-genome are found in less than one sixth of strains sequenced (Livingstone et al., 2018c). These observations suggests that the composition of the cocktail of secreted toxins can vary considerably between individual strains, which in turn could explain the variability in predatory activity against specific prey exhibited by phylogenetically similar myxobacteria (Livingstone et al., 2017).

In only a few specific cases, have proteins or metabolites been identified which are responsible for predation of particular prey by myxobacteria, or the resistance of prey to myxobacterial attack (DePas et al., 2014; Müller et al., 2014, 2015; Pérez et al., 2014). We therefore want to identify the proteins/metabolites secreted by myxobacteria which are responsible for predatory activity, both generally and prey-specifically. As predatory secretions seem to be strain-specific patchworks of proteins and metabolites, and if we assume that predatory activity against a particular prey organism is likely to be due to one or two key gene products, genome-wide association studies (GWAS) may have the potential to identify the genes responsible for myxobacterial predatory activity.

Genome-wide association studies assess whether phenotypic variation can be correlated with genetic differences, in order to identify genes responsible for causing the phenotypic variation. Traditionally, GWAS has been applied to humans, which have extremely similar genomes to one another when compared to myxobacterial species. Genetic variations investigated in humans have therefore been single-nucleotide polymorphisms, rather than gene presence/absence. The first example of bacterial GWAS was published in 2010 (van Hemert et al., 2010), and the majority of studies published since then have almost exclusively focused on pathogens, investigating binary phenotypes subject to strong selective pressures such as drug resistance (Power et al., 2017). Pathogens are also under strong selective pressure to grow fast, and lose non-beneficial genes quickly, which might be expected to be a requirement for successful GWAS. A notable early application of GWAS across bacteria, screened for genes that were found in predators but that were absent from non-predators, identifying genes associated with a predatory lifestyle, including the mevalonate pathway for isoprenoid biosynthesis (Pasternak et al., 2013).

We wished to assess whether a GWAS approach could identify associations between predatory activity and myxobacterial gene presence/absence, to provide shortlists of candidate genes likely to be involved in predation. Could GWAS cope with diverse genotypes, phenotypes with relatively weak selective pressures, and for organisms which seem under little pressure to streamline their genomes compared to pathogenic bacteria?

## Materials and Methods

### Genome Sequences

General features of the 29 genome sequences used in this study are presented in Table 1. Nineteen genomes belonged to the genus *Corallococcus*, and have been published elsewhere (Livingstone et al., 2018c). The other ten were from *Myxococcus* spp., including one that has been described previously (*Myxococcus xanthus* DK1622; Goldman et al., 2006). The remaining nine *Myxococcus* spp. genome sequences were obtained for this study using the MicrobesNG service, described by Livingstone et al. (2018c) and are available at GenBank under the following Genome accession numbers: VHLD00000000 (AB022), SRLY00000000 (AB024B), SRLX00000000 (AB0-2 5A), SRLW00000000 (AB025B), VHLC00000000 (AB036A), VHLB00000000 (AB056), SRLV00000000 (CA005), SRLU00 000000 (CA006), and VHLA00000000 (CA010).

**TABLE 1:** General features of the genome sequences used in this study.

**Strains**	**Taxonomy**	**Size (bp)**	**% GC**	**CDS**	**N50**	**L50**	**Coverage**	**‘Predatory breadth’**	**Description**
AB004	*Corallococcus* spp.	10,604,304	69.4	8,023	26,897	119	35.2	0/10	Livingstone et al., 2018c
AB018	*Corallococcus* spp.	10,463,000	69.4	8,544	37,048	94	49.2	4/10	Livingstone et al., 2018c
AB022	*Myxococcus* spp.	9,062,526	68.9	7,498	69,990	38	159.9	1/10	This study
AB024B	*Myxococcus* spp.	9,058,989	68.9	7,034	47,907	55	51.2	1/10	This study
AB025A	*Myxococcus* spp.	9,051,712	68.9	7,002	45,473	60	50.4	1/10	This study
AB025B	*Myxococcus* spp.	10,601,221	70.1	8,173	27,997	121	80.1	4/10	This study
AB032C	*Corallococcus* spp.	10,451,389	69.5	7,976	73,011	43	127.0	1/10	Livingstone et al., 2018c
AB036A	*Myxococcus* spp.	9,273,113	69.0	7,269	72,065	41	93.5	3/10	This study
AB043A	*Corallococcus* spp.	10,150,784	70.3	7,664	19,246	164	40.8	1/10	Livingstone et al., 2018c
AB045	*Corallococcus coralloides*	9,940,502	69.9	7,707	37,416	80	76.2	0/10	Livingstone et al., 2018c
AB047A	*Corallococcus* spp.	9,538,803	69.8	7,416	54,870	51	164.7	4/10	Livingstone et al., 2018c
AB049A	*Corallococcus* spp.	9,526,569	69.7	7,224	17,173	170	33.6	3/10	Livingstone et al., 2018c
AB050A	*Corallococcus* spp.	9,983,374	70.0	8,267	29,422	115	56.9	1/10	Livingstone et al., 2018c
AB050B	*Corallococcus* spp.	9,397,643	70.1	7,852	42,207	68	99.2	6/10	Livingstone et al., 2018c
AB056	*Myxococcus* spp.	9,106,805	69.0	7,098	75,794	37	130.1	5/10	This study
CA005	*Myxococcus* spp.	9,110,392	68.9	7,205	81,131	35	60.6	0/10	This study
CA006	*Myxococcus* spp.	9,046,377	68.9	7,010	46,018	64	60.6	0/10	This study
CA010	*Myxococcus* spp.	9,046,872	68.9	7,005	68,574	46	111.7	5/10	This study
CA031B	*Corallococcus* spp.	10,514,033	69.6	7,670	20,241	151	74.2	4/10	Livingstone et al., 2018c
CA040B	*Corallococcus* spp.	10,401,616	70.2	7,618	26,404	119	118.2	3/10	Livingstone et al., 2018c
CA041A	*Corallococcus* spp.	10,265,543	69.5	7,859	30,701	110	82.3	0/10	Livingstone et al., 2018c
CA043D	*Corallococcus* spp.	10,794,417	69.9	8,319	37,418	80	67.3	10/10	Livingstone et al., 2018c
CA047B	*Corallococcus* spp.	10,336,837	69.9	7,717	25434	123	81.4	10/10	Livingstone et al., 2018c
CA049B	*Corallococcus coralloides*	9,633,170	70.2	7,368	34676	82	37.2	9/10	Livingstone et al., 2018c
CA051B	*Corallococcus* spp.	10,527,286	70.3	7,720	15085	220	30.6	3/10	Livingstone et al., 2018c
CA053C	*Corallococcus* spp.	10,518,560	70.1	7,911	20162	153	54.2	2/10	Livingstone et al., 2018c
CA054A	*Corallococcus* spp.	10,352,759	69.5	7,695	24469	134	66.8	0/10	Livingstone et al., 2018c
CA054B	*Corallococcus coralloides*	9,916,432	70.0	7,671	39232	80	47.8	10/10	Livingstone et al., 2018c
DK1622	*Myxococcus xanthus*	9,139,763	68.9	7,181	n/a	n/a	–	0/10	Goldman et al., 2006

*Predatory breadth is the number of different prey organisms for which the strain was amongst the top third most efficient predators.*

### Predation Assays

Predation assays were undertaken using the method of Livingstone et al. (2018c), with *Escherichia coli*, *Pseudomonas aeruginosa*, *Klebsiella pneumoniae*, *Proteus mirabilis*, *Staphylococcus aureus*, *Staphylococcus epidermidis*, *Staphylococcus saprophyticus*, *Enterococcus faecalis*, *Bacillus subtilis*, and *Candida albicans* as prey organisms. Myxobacteria and prey organisms were cultivated as described by Livingstone et al. (2017). Myxobacterial cell suspensions were spotted onto lawns of each prey organism, and the diameter of the resulting zones of predation measured after 7 days. Data for *Corallococcus* spp. strains has been published previously (Livingstone et al., 2018c), but the data for *Myxococcus* spp. strains was generated for this study. Data from 7 days’ incubation was used instead of the 4 day data reported previously (Livingstone et al., 2017), to maximise differences between the most efficient and least efficient predators.

To test the effects of formaldehyde mitigation, predation assay plates were supplemented with filter-sterilised formaldehyde dehydrogenase (Fdh) at 12.5 mU/ml and 1 mM NAD^+^. Fdh from *Pseudomonas* sp. and NAD^+^ were both obtained from Sigma-Aldrich, United Kingdom.

### GWAS Analysis

Putative proteins encoded by the 29 myxobacterial genome sequences were allocated to clusters of orthologous proteins by Roary, with the default ‘number of clusters’ parameter changed to 200,000 to allow for divergent genomes (Page et al., 2015). Roary is a tool for pan-genome analysis, identifying common genes shared by multiple genomes. Orthologous clusters were then associated with predation phenotypes using Scoary (Brynildsrud et al., 2016), which identifies associations between phenotypes of strains and their complements of genes. For inputting into Scoary predators were defined as ‘superior,’ ‘inferior,’ and ‘moderate’ predators, for each prey organism. For each prey the myxobacteria were ranked by their predatory activity (diameter of predatory zone in the predation assays) and the top third (typically nine) predatory strains were defined as being ‘superior’ predators, the bottom third (typically nine) were defined as ‘inferior’ predators, with the remainder (typically eleven) considered ‘moderate’ predators (with precise numbers depending on the granularity of the predation dataset for each prey organism). For Scoary analysis, phenotypic values were defined as ‘1’ for superior predators, ‘0’ for inferior predators and no phenotypic value was assigned for moderate predators. Scoary’s *p*-value cut-off was set to <0.05, the sensitivity cut-off was set to 70% and specificity to 80%, so that orthologous clusters associated with predation would typically be found in at least 7/9 ‘superior’ predators, or be absent from all but 1/9 ‘inferior’ predators. These values were chosen to give an approximate number (tens) of genes per prey organism considered optimal for downstream experimental investigation, and took into consideration that we expected multiple predatory mechanisms to be in operation within each strain – i.e., possession of a ‘superior’ predatory gene would make any strain a ‘superior’ predator, but strains could be ‘superior’ predators without that gene if they possessed alternative predatory genes.

## Results

### Myxobacterial Genomes and Predation Phenotypes

For this study, 29 myxobacterial strains from two genera were chosen for analysis: *Corallococcus* spp. and *Myxococcus* spp. are the most commonly isolated myxobacteria from terrestrial soils (Mohr et al., 2016; Livingstone et al., 2017). Nineteen organisms were *Corallococcus* spp. while ten were *Myxococcus* spp., reflecting their relative abundance in soil and culture collections, and we also included the single best characterised myxobacterium (*Myxococcus xanthus* DK1622). All other strains were from a collection of isolates described previously (Livingstone et al., 2017). For each strain predatory activity was determined as the diameter of a zone of killing observed by inoculating myxobacteria onto lawns of ten prey organisms (Gram-negative bacteria, Gram-positive bacteria and a yeast), and are provided as Supplementary Table S1. Strains were selected for inclusion in this study to give a set exhibiting diverse predatory phenotypes.

**FIGURE 1:**
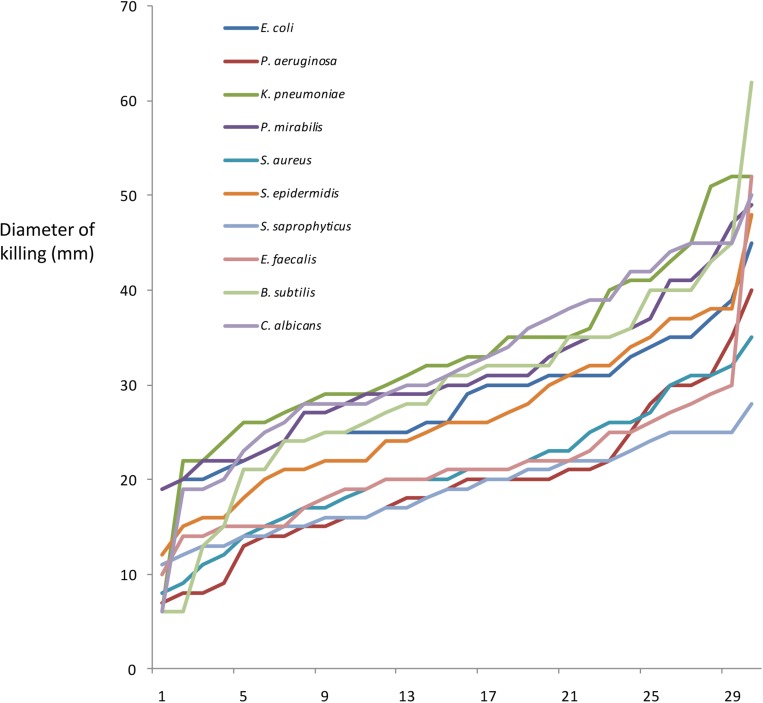
Predation efficiency (mm diameter of predation zone after 7 days), of 29 predatory strains, when preying upon 10 prey organisms. For each prey organism, strains are arranged in order of increasing predation efficiency.

To perform GWAS, Roary was used to sort myxobacterial gene products into orthologous clusters (Supplementary Figure S1). Then Scoary was used to assess any correlation between the presence/absence of cluster members with phenotype. Scoary requires phenotypic data to be binary; however, all myxobacteria isolates are predatory, albeit exhibiting different efficiencies of predation. Figure 1 shows the ordered predation efficiency of the 29 predators preying upon each prey organism. While occasional isolates show unusually low or high predatory efficiency, most isolates have very similar phenotypes. When using Scoary we therefore ranked myxobacteria by their predatory activity against each prey organism and defined the top third as being ‘superior’ predators, the bottom third as ‘inferior’ predators, and the remainder as ‘moderate’ predators. We also obtained a pan-prey overview of predatory efficiency, by ranking predators according to the number of prey against which they were considered to be ‘superior’ predators, a metric we call ‘predatory breadth’ in Table 1.

### Predatory Efficiency Correlates With the Presence/Absence of Multiple Gene Clusters

Scoary was used to filter gene clusters associated with predatory activity against each prey organism (Supplementary Table S2). 139 putative ‘predation genes’ were identified, most of which were associated with predation activity against more than one prey. 62/139 genes were associated with predatory activity against single prey, 73/139 were associated with predatory activity against two or three prey, 1/139 (encoding a hypothetical protein), was associated with predatory activity against five prey, while 1/139 (encoding CTP synthase) was associated with predatory activity against six prey.

**FIGURE 2:**
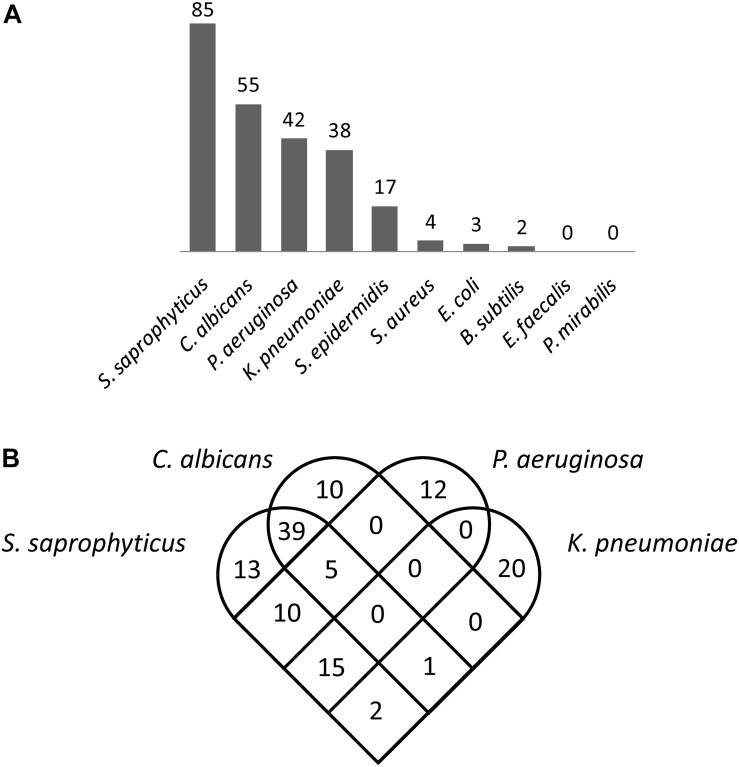
Myxobacterial genes associated with superior predation. **(A)** The number of predatory genes identified for each of the 10 prey organisms tested. **(B)** Myxobacterial genes associated with superior predation of four prey organisms (the four for which the largest numbers of myxobacterial genes were identified).

The numbers of genes associated with predatory activity varied substantially between different prey organisms (Figure 2), with 85 genes identified for *S. saprophyticus*, 55 for *C. albicans*, 42 for *P. aeruginosa*, 38 for *K. pneumoniae*, 17 for *S. epidermidis*, four for *S. aureus*, three for *E. coli*, two for *B. subtilis*, and none for *E. faecalis* and *P. mirabilis*. Figure 2 shows the overlap in genes associated with predatory activity against the four organisms with the largest numbers of genes identified. The greatest overlaps in genes identified were between *S. saprophyticus* and *C. albicans* (45 genes) and between *S. saprophyticus* and *P. aeruginosa* (30 genes), however, only five genes were associated with predatory activity against both *P. aeruginosa* and *C. albicans*. This was unexpected, as *C. albicans* is eukaryotic, while *S. saprophytics* is a Gram-positive bacterium and *P. aeruginosa* is a Gram-negative bacterium. These observations suggest that there are very few, if any, genes that are universally required for predatory activity regardless of prey phylogeny, and that the genes which confer predatory activity tend to be particularly beneficial against multiple prey from profoundly different taxa, with little correlation between phylogeny of prey and the set of predatory genes identified.

For 99 of the 139 genes, EggNOG mapper was able to allocate the encoded protein to a COG functional category (Huerta-Cepas et al., 2017), allowing assessment of whether the ‘predatory protein’ set was enriched in particular functional categories. However, the frequency profile of COG category membership was not found to be significantly different from that of the parent genomes (*p* > 0.05). For instance, of the 139 predatory genes identified, 64 (46%) encoded hypothetical proteins, a similar proportion to that of hypothetical genes in a typical myxobacterial genome (∼41%). Of the remaining 75 predicted proteins with annotations, some commonalities were observed. Ten are putative transcriptional regulators and another seven proteins are associated with kinase signalling (Ser/thr kinases and two-component system histidine kinases). Five proteins are likely involved in FA/lipid metabolism, six are transporters or secretory proteins, and five are suggested to be involved in DNA modification (including recombinases, restriction/modification enzymes and helicases).

### Formaldehyde Dismutase Is Associated With Effective Predation of *Pseudomonas aeruginos*a

Some of the predation genes identified above had annotations which immediately suggested a plausible mechanism for predatory activity. For instance, screens against *S. saprophyticus*, *P. aeruginosa* and *K. pneumoniae* identified genes encoding a polyketide synthase (*ppsE*) and a penicillin-binding protein. One such protein’s annotation (formaldehyde dismutase, Fdm, which catalyses the conversion of formaldehyde into formate and methanol), also immediately suggested a mechanism which was amenable to simple testing. The *fdm* gene was associated with superior predation of *P. aeruginosa* and *C. albicans*, but was not associated with superior predation of any other prey organisms tested. *P. aeruginosa* is known to secrete toxic formaldehyde, and to produce its own formaldehyde de-toxifying enzymes, such as Fdh which converts formaldehyde into formate (Willsey and Wargo, 2015). Therefore we hypothesised that myxobacteria lacking the *fdm* gene were inhibited by formaldehyde secreted by prey *P. aeruginosa*, and that possession of *fdm* protected predatory strains from the secreted formaldehyde, allowing them to predate more efficiently.

**FIGURE 3:**
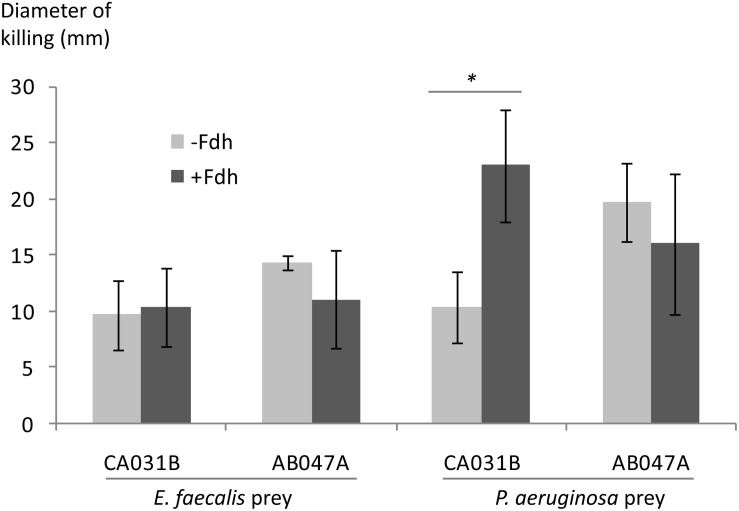
Efficiency of predation by myxobacteria possessing (AB047A) and lacking (CA031B) the *fdm* gene, in the presence and absence of formaldehyde dehydrogenase (±Fdh), with *P. aeruginosa* and *E. faecalis* prey. Predatory efficiency is expressed as the diameter of the zone of prey killing 7 days after predator was spotted onto a lawn of prey organism. Errors bars represent ±1 standard deviation, *n* = 3. Significant differences (*p* < 0.05) indicated with an asterisk.

To test this hypothesis, predatory activity was tested for predator strains possessing and lacking the *fdm* gene, against *P. aeruginosa* and *E. faecalis* prey. Assays were replicated in the presence and absence of Fdh (and its cofactor NAD^+^), which detoxifies formaldehyde into less toxic formate. Figure 3 shows the results of those assays, with predatory efficiency indicated by the size of the zone of prey-killing when predator is spotted onto lawns of prey. Addition of Fdh to the medium significantly (*p* < 0.05) enhanced the predatory activity of a strain lacking *fdm* (CA031B), but not that of a strain possessing *fdm* (AB047A). No enhancement of predatory activity was observed by Fdh addition when *E. faecalis* was used as prey (Figure 3). This suggests that formaldehyde secreted by *P. aeruginosa* does inhibit predators, and that efficient predators have evolved a mechanism to detoxify formaldehyde secreted by prey.

## Discussion

Microbial competition can be likened to an arms race, with predators repeatedly evolving new mechanisms of attack, as prey evolve to resist those mechanisms and develop their own counter-attacks. As the conflict continues, outmoded attack mechanisms no longer provide a benefit and are lost as genomes streamline themselves through adaptive gene loss (Bosi and Mascagni, 2019). Myxobacterial predators have the largest genomes of any bacterial order, and share genes extensively through a large accessory pan-genome, which includes the genes for digestive enzymes and secondary metabolites (Livingstone et al., 2018c). Although all myxobacteria have a broad prey range, Individual strains exhibit different efficiencies of predation when consuming particular prey, which is likely due to the unique set of predatory genes each strain possesses (Livingstone et al., 2017).

These phenomena suggest that the genes responsible for efficient predation on a particular prey should be relatively enriched in those strains which exhibit efficient predation of that prey, and be relatively impoverished in strains exhibiting poor predatory activity against that prey. It could also suggest that the complement of predatory genes present in a strain’s genome is indicative of the organisms that strain has historically preyed upon.

### Genomic and Phenotypic Inputs to GWAS

Several aspects of our phenotypic and genomic input data were non-ideal for GWAS, having the potential to reduce the strength of gene-phenotype association in various ways. We therefore took a straightforward computational approach, and applied low-stringency filters to screen GWAS output.

The phenotypic input data used here had to be made binary for input into Scoary. To give separation between the best and worst predators, the set of predators was divided as closely as possible into thirds and the top third defined as superior predators (‘1’), the bottom third as inferior predators (‘0’), and the remainder as moderate predators (‘NA’). Defining larger proportions of the predators as moderate predators would have provided greater separation of superior from inferior predators, however, it would also have reduced the number of superior and inferior predators filtered by Scoary, weakening the strength of the analysis or increasing the number of false positives.

This study used a small number of diverse genomes, spanning the diversity of two genera. This provided a great deal of phenotypic variation, but resulted in a relatively small number of genes that were shared enough to have the potential to provide a positive identification. For instance, accessory pan-genome genes are typically found in only a small proportion of genomes even within a single genus (Livingstone et al., 2018c), but the Scoary analysis required at least 70% of superior predator strains to possess an orthologue to give a high enough sensitivity for identification. Using a set of more similar genomes would increase the ratio of core:accessory genes in the analysis, expanding the number of genes considered. Such a reduction in organism diversity might not even reduce observed phenotypic variation, as predatory phenotypes are known not to be congruent with phylogeny in myxobacteria (Livingstone et al., 2017). In addition, with the exception of *M. xanthus* D1622, the genomes used here were all draft genomes, allowing the potential for important predation-associated genes to be missing from the genome sequences. This was compensated for by setting Scoary cut-offs which allowed genes to be associated with predation, even if they were missing from one or two ‘superior predator’ genomes.

Myxobacterial predatory activity does not correlate with phylogeny, varies depending on which prey organism is being preyed upon, and predatory genes are enriched in the accessory rather than core pan-genome (Livingstone et al., 2017, 2018c). We would therefore expect killing of different prey by particular myxobacterial strains to be a consequence of a nearly unique combination of multiple predation genes, many acquired horizontally. This hampers detection of genotype-phenotype associations by GWAS, as the requirement for orthologues to be present in the majority of ‘superior predators,’ means that genes from the large accessory pan-genome were essentially undetectable. This potentially explains why only one polyketide synthase gene was identified by GWAS, despite myxobacterial genomes being renowned for their large numbers of secondary metabolite biosynthetic gene clusters and their likely involvement in predation.

### GWAS Produced a Set of Candidate Genes for Further Study

Nevertheless, despite the potential pitfalls discussed above, GWAS was able to identify practical numbers of predation-associated genes. We would expect some of the predation genes identified to be false positives, for example, tRNA-amino acyl ligases – given their housekeeping roles. However, other proteins could easily be imagined to be important for predation, including transporters for secreting toxins or for taking up prey-derived nutrients. Many of the identified proteins were hypothetical proteins with no annotated function, raising the exciting possibility of them being novel antimicrobial proteins.

Very different numbers of predation genes were identified for particular prey organisms. No genes at all were identified for *E. faecalis* or *P. mirabilis*, while *S. saprophyticus* had 85 genes identified. However, other species in the *Staphylococcus* genus had only 17 and four genes identified (*S. epidermidis* and *S. aureus*, respectively) using this approach. For the genes identified as being associated with predation of more than one prey organism, the prey were usually from completely different taxa [for example the greatest overlap in predation genes identified was between *C. albicans* (a yeast) and *S. saprophyticus* (a Gram-positive bacterium]. The number of genes identified therefore doesn’t seem to relate to prey phylogeny, nor can it be explained by variability in the predatory phenotypes of the different prey. This might reflect the taxonomic distribution of the molecular targets of predation genes, which could potentially be different from prey phylogeny, or it may be a consequence of varying likelihoods of false-positive gene identification for different prey.

Molecular investigations into individual genes can be extremely time-consuming (much more so than isolation and genome sequencing), so for any given sensitivity and specificity it would be useful to know how many genomes are required in order to obtain a certain (small and focused) number of target genes. The relationship between the number of genomes included and the resulting number of genes identified by GWAS can be used to predict how many genome sequences will be required in order to produce a given number of correlated genes. Repeating the GWAS analysis with varying sized subsets of the genomes gave a curve showing the relationship between the number of identified genes as a function of the number of genomes included. The resulting curve could be fitted to a power law distribution (the number of genes identified = 22601 × *G*^–1.4^, where *G* is the number of genomes). Using this relationship it could be predicted that 48 genome sequences of similar diversity would be required to produce less than 100 target genes, while 250 genome sequences would be required to reduce the target gene list to 10 candidate genes. Of course adding in more genomes to get fewer genes would also depend on relatedness of organisms included and the diversity of gene sets within their genomes. Effects of variables such as these would benefit from systematic investigation when more genome sequences become available.

### GWAS, Genome Streamlining, and Life History Traits

Genome-wide association studies are predicated upon the concept that genes can be gained/lost, and that the current gene set in an organism’s genome is indicative of its life history traits. This perspective assumes that the contemporary gene set is composed of genes with function (albeit maybe subtle), with fast conversion of non-advantageous genes into pseudogenes and purging from the genome. This ‘selectionist’ perspective tends to dominate the bacterial physiology literature, with authors often assuming implicitly that genes found in the genome are functional and confer a selectable advantage. This viewpoint has probably become adopted because of the genomic streamlining exhibited by the model bacteria whose genomes were the first to be sequenced and compared. However, bacterial genomes subject to streamlining tend to be small, with low % GC content (Giovannoni et al., 2014; Almpanis et al., 2018). With the largest bacterial genomes, and around 70% GC content, myxobacteria would seem to not to be subject to such streamlining selection. Therefore, rather than assuming that the large myxobacterial gene set provides subtle selective advantages under very specific ecological situations, it seems more parsimonious to suggest that the slow growth rate of myxobacteria insulates them from streamlining selection, allowing them to accumulate a patchwork of predatory genes.

Our GWAS analysis suggests predation genes are largely prey-specific, but presumably the lack of streamlining selection means that they can be retained in myxobacterial genomes without the need for frequent or recent selection by growth on that prey organism. The myxobacterial predatory mechanism of constitutive secretion of cocktails into a public commons might also reduce the strength of any selection acting on individual (competing) strains sharing a locale.

The broad prey range of myxobacterial predation is thus likely to be a consequence of the accumulation of sets of prey-specific predation genes, rather than possession of a set of ‘generally antimicrobial’ genes. If this proposal is correct, it undermines the original rationale for applying GWAS to myxobacterial predation, but regardless, the GWAS approach was able to provide valuable insights into predatory mechanisms. We assume that although the streamlining selection is weak in myxobacteria, that there is still enough to allow for enrichment in the genome of predation genes with only infrequent exposure to sensitive prey, reducing but not abolishing any GWAS signal.

### Pseudomonas Formaldehyde Secretion

*Pseudomonas aeruginosa* makes formaldehyde as an intermediate when catabolising sarcosine, requiring expression of a formaldehyde detoxification system (Willsey and Wargo, 2015). *Candida* spp. including *C. albicans*, have also been shown to produce and degrade formaldehyde (Sakai and Tani, 1988; Tsai et al., 2005; Tillmann et al., 2015). Fdm in myxobacteria was associated with superior predation of *P. aeruginosa*, and we therefore hypothesised that formaldehyde secreted by *P. aeruginosa* and *C. albicans* poisons myxobacterial strains lacking Fdm, impeding their ability to predate efficiently. In support of this hypothesis, addition of Fdh increased the predatory activity of myxobacteria lacking Fdm, in a prey and strain-specific fashion (Figure 3).

Nevertheless further experimental investigation is needed, to further challenge the hypothesis and to establish any ecological relevance. To establish causality, ideally, experiments would transplant the *fdm* gene from a superior predator into an inferior predator and see whether that increases predatory activity. Similarly, deletion of *fdm* would be expected to reduce predatory activity against *P. aeruginosa*. Unfortunately, genetic engineering of myxobacteria is not straightforward, although the myxobacteria genetic manipulation toolkit is gradually expanding. Such experiments would ideally be complemented by engineering prey mutants which produce more and less formaldehyde, and testing their effects on predation efficiency. What concentrations of formaldehyde does *P. aeruginosa* secrete? Would addition of formaldehyde at that concentration to a non-secreting prey hinder predation by myxobacteria lacking *fdm*?

Maybe formaldehyde production by *P. aeruginosa* evolved as an anti-predation adaptation. Or perhaps, like the spandrels of San Marco (Gould and Lewontin, 1979), formaldehyde secretion evolved for another (metabolic?) reason, with its secondary antimicrobial activity being enhanced by evolution subsequently. This would mirror myxobacterial evolution, where constitutively secreted OMVs were selected by evolution to carry toxic proteins and metabolites, enhancing their predatory activity (Whitworth, 2011).

### Concluding Comments

In this study, we used bacterial GWAS to investigate the predation of ten prey organisms by diverse myxobacteria. Myxobacterial genes were identified whose presence/absence was associated with efficient predation, and experimental validation confirmed one such association. This study applies bacterial GWAS to a non-pathogenesis phenomenon, and demonstrates that GWAS can be successfully applied to diverse environmental organisms which share a common phenotypic trait. We also propose that the broad prey range of myxobacteria is due to myxobacterial genomes accumulating large numbers of prey-specific predation genes, rather than evolution selecting for strains carrying broad-range predatory genes.

## Data Availability Statement

The datasets generated for this study can be found in GenBank under the following genome accession numbers: VHLD00000000 (AB022), SRLY00000000 (AB024B), SRLX00000000 (AB025A), SRLW00000000 (AB025B), VHLC00000000 (AB036A), VHLB00000000 (AB056), SRLV00000000 (CA005), SRLU00000000 (CA006), and VHLA00000000 (CA010).

## Author Contributions

DW conceived the study, and drafted the manuscript, which all authors edited. DS performed the GWAS and comparative genomics analyses, supervised by DW and MS. PL coordinated the genome sequencing and deposition of sequences in GenBank. EF performed the assays investigating *fdm* and formaldehyde.

## Conflict of Interest

The authors declare that the research was conducted in the absence of any commercial or financial relationships that could be construed as a potential conflict of interest.
