# Supplementary figures and images for: Genome-Wide Identification of Myxobacterial Predation Genes and Demonstration of Formaldehyde Secretion as a Potentially Predation-Resistant Trait of Pseudomonas aeruginosa
Source: Front Microbiol. 2019 Nov 13;10:2650. doi: 10.3389/fmicb.2019.02650 (PMC6863768; doi:10.3389/fmicb.2019.02650)

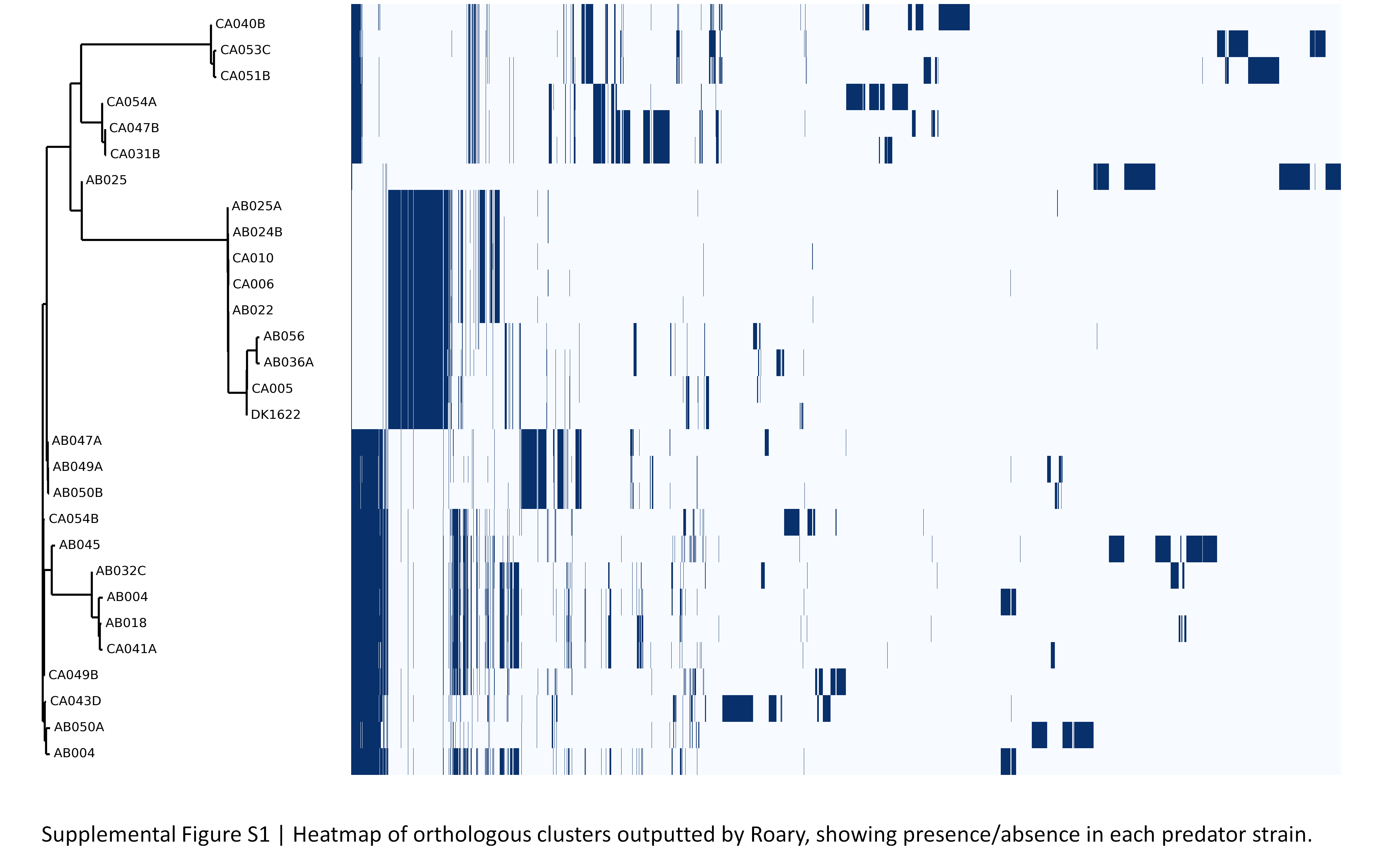

Supplement: Supplementary file 3 [file Image_1.jpg]
